# Food neophobia: psychological dimensions of consumer perception and emotional sentiment in social media discourse

**DOI:** 10.3389/fnut.2025.1584409

**Published:** 2025-06-26

**Authors:** Yu Shan, Hong Wang, Wenqi Wang

**Affiliations:** ^1^School of Management Science, Chengdu University of Technology, Sichuan, China; ^2^School of Business, Chengdu University of Technology, Chengdu, Sichuan, China

**Keywords:** future foods, perception of future foods, neophobia, social media analytics (SMA), Latent Dirichlet Allocation (LDA)

## Abstract

Addressing global food security necessitates exploring future foods, yet their societal acceptance hinges critically on public perception an2d psychological barriers such as neophobia. This study delves into the psychological dimensions underlying consumer perception of future foods, investigating the intricate relationship between food neophobia and these perceptions, and mapping the prevailing emotional landscape surrounding novel food adoption. Employing a Social Media Analytics (SMA) framework to capture ecologically valid public discourse, we utilized social media text analysis, integrating topic modeling and sentiment analysis, to dissect online expressions concerning future foods. Our analysis reveals that public evaluations are predominantly positive (53.20%), while a substantial segment expresses negative sentiments (30.48%) and ambivalence (16.32%). Psychologically, we identified four salient perceptual dimensions – taste, appearance, culture, and technology – which differentially mediate food neophobia and elicit distinct emotional valences. Notably, appearance and cultural perceptions are associated with heightened neophobia and negative emotional responses, suggesting underlying psychological mechanisms of sensory and socio-cultural rejection. These findings offer critical psychological insights for future food producers and policymakers, highlighting the psychological determinants of public attitudes toward future foods and informing psychologically-informed strategies to enhance consumer acceptance and promote dietary innovation.

## 1 Introduction

The advent of future foods, driven by cutting-edge technologies like artificial intelligence, gene editing, and synthetic biology, is envisioned as a transformative shift in food production (1). While offering promising solutions to global food security and supply challenges, the psychological landscape of public perception remains a critical determinant of acceptance for these novel foods. Specifically, underlying psychological processes, such as food neophobia – the inherent reluctance to consume new foods – and associated emotional responses, play a pivotal role in shaping consumer attitudes. In China, the future food market is experiencing rapid growth, alongside this burgeoning market, understanding how the predominantly public perceives these novel food products and technologies is critical. This research, therefore, adopts a Social Media Analytics (SMA) approach to explore these psychological dimensions of public perception. By analyzing key domains of public concern expressed online, this study aims to provide psychological insights into consumer attitudes toward future foods, ultimately informing strategies to foster greater market acceptance and facilitate the integration of these innovations into dietary practices.

### 1.1 Future food

Beyond these technological underpinnings, the concept of future foods also encompasses a wide array of novel protein sources and production methods aimed at addressing global food security and sustainability challenges (2, 3). This includes exploring alternative protein sources like insects (4–7), microalgae (8), and the development of cultured meat (3, 9) and plant-based alternatives. Such innovations represent a significant departure from traditional agriculture and food systems, requiring new approaches to production, regulation, and consumer acceptance (10).

Unlike traditional foods, future foods are produced rather than grown, providing them with unique advantages in food supply (11, 12). The concept of future foods has garnered significant attention and recognition within both academia and industry. Discussions on social media further reveal the public’s growing interest in future foods (13, 14). For instance, the Zhihu topic “Artificial meat becoming popular in China, yet some disdain such ‘fake meat’ “accumulated 4,565,443 views within 3 months, while “Algae-modified food might become the next new food” gathered 239,441 views in the same period. Additionally, future foods appeared 37 times in the top 50 trending topics on Weibo between 2022 and 2023, with a cumulative trending time of 242 h.

### 1.2 What is the perception of future food?

Despite the widespread attention and unique advantages of future foods, research indicates that individuals may exhibit resistance toward these novel foods (5, 15). Building upon existing research that has identified key factors influencing future food acceptance, such as neophobia and sensory properties (16, 17), this study aims to provide a novel, data-driven exploration of public perceptions as expressed in real-world social media discourse, offering a more ecologically valid understanding of the multifaceted nature of these perceptions and their relationship with neophobia and emotional responses. Public apprehension toward future foods like lab-grown meat often stems from fundamental concerns surrounding unfamiliarity and a perceived lack of naturalness (18). The rapid pace of technological advancement in food production outpaces public understanding, creating a knowledge gap exploited by misinformation. Historical precedents of food safety concerns and a deep-seated preference for traditional, recognizable food sources further contribute to distrust (15). This inherent fear of the unknown, coupled with anxieties about long-term health impacts and ethical implications, forms the core basis of consumer neophobia, presenting a significant challenge for fostering acceptance and integration of these novel food technologies into the mainstream market. Identifying the perceptual factors underlying future foods is critical for their production and marketing. Previous studies have found that food neophobia—reluctance to consume foods produced using new technologies—is common among the public (14, 16). Moreover, the appearance of new foods may trigger visual stimulation and socio-cultural conflict, leading to psychological rejection (17, 19, 20). The taste and texture differences between new and traditional foods can also result in initial sensory sensitivity, causing physiological aversion (18, 21). These fears and anxieties diminish consumers’ willingness to try and purchase new foods (22–24). As such, as a typical example of new foods, public perceptions of future foods significantly influence their market acceptance and promotion (45).

### 1.3 Application of SMA in future food

To address these challenges, social media plays a vital role in the discussion and promotion of future foods. Social media platforms offer an open space for consumers to share their views and experiences, thereby enhancing public awareness and understanding of future foods (25). By actively leveraging social media, researchers and businesses can better understand consumer needs and concerns (26), allowing for the adjustment of products and marketing strategies to improve market acceptance of future foods. In summary, while future foods hold significant potential in addressing food security issues and diversifying food sources (1), understanding public perceptions through social media is crucial for increasing their acceptance.

Previous research has confirmed that social media effectively reflects the thoughts of the majority (26). This study aims to explore public perceptions of future foods using social media data, employing Social Media Analytics (SMA) for the analysis. Through descriptive analysis of social media data, the study investigates public attention trends toward future foods, utilizing the LDA framework for topic modeling to identify four key dimensions of public concern on social media regarding future foods. Additionally, the word embedding analysis method is employed to explore the relationship between different perceptual dimensions of future foods and neophobia. The TF-IDF algorithm is further used to extract important keywords and explore emotional orientations toward future foods. Finally, emotional orientations under each of the four dimensions are examined to understand the emotional evaluation status of future foods across different dimensions.

This study using SMA expands the understanding of future food perception, laying the groundwork for subsequent research. By analyzing social media data, the study provides a more accurate understanding of public perception and neophobic of future foods. Offers precise insights into the perception and emotional states across different dimensions of future foods, providing theoretical guidance for food development and marketing.

## 2 Materials and methods

**Figure 1:**
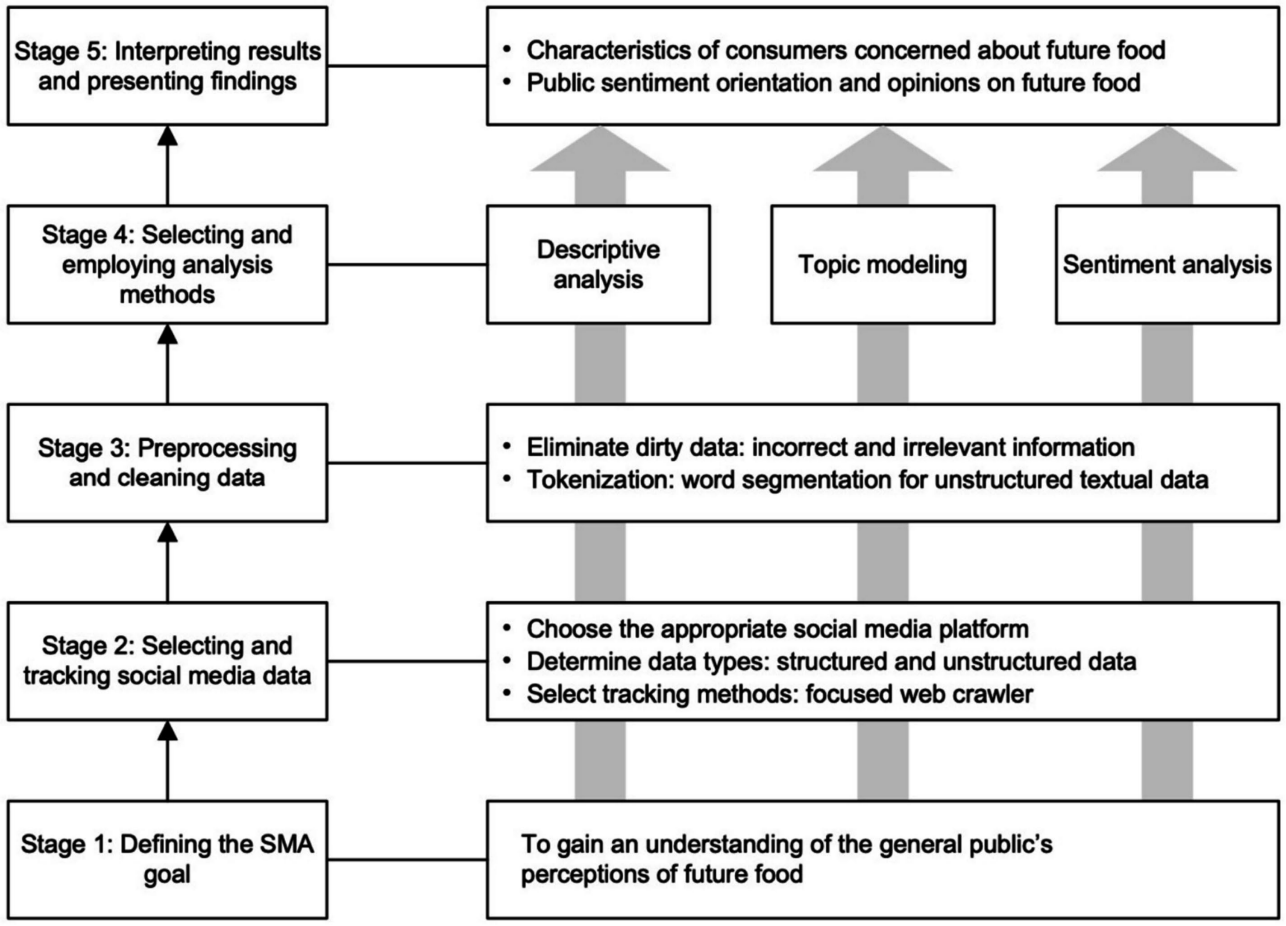
An overview of the five-stage SMA framework used in the study.

Based on the five-stage framework of SMA, this study involves the collection of social media data and the determination of data processing methods to understand public perceptions of future foods. Subsequently, by analyzing each perceptual state, the study aims to comprehend the emotional states associated with different perceptions of future foods. Following the widely adopted five-stage SMA framework (identifying research objectives, data collection, data preprocessing, analysis, and interpretation), this study systematically investigates public discourse on future foods. This framework guides the systematic collection, processing, analysis, and interpretation of social media data to extract meaningful insights into public attitudes. Specifically, the research begins with defining clear objectives, followed by the systematic collection of relevant data from social media platforms, rigorous preprocessing to ensure data quality, subsequent analysis using appropriate techniques, and finally, the interpretation of findings in the context of the research questions. The structural flowchart of the methodology, highlighting these key SMA stages as implemented in our research, is illustrated in Figure 1.

### 2.1 Data collection

After identifying the research subjects, the first step in our SMA framework is to select the data sources, specifically social media platforms. Considering the widespread use of these platforms and the frequency of discussions about future foods, we collected textual information from representative websites such as “Food Partner Network” and “Catering Industry Forum.” Additionally, we scraped text data from 30 relevant topics on Weibo, including #FutureFoods# and #WhatDoFutureFoodsTasteLike#. These 30 topics were selected based on a preliminary exploration of Weibo’s trending topics and search results related to future foods and related concepts (e.g., artificial meat, plant-based protein, lab-grown meat, novel food technology) during the initial phase of data collection. We identified topics that were consistently popular, generated significant discussion volume, and directly addressed aspects of future foods, aiming to capture a broad and representative sample of public discourse on the platform. Other platforms like Zhihu (a major Chinese question-and-answer platform) and Bilibili (a popular Chinese video sharing website with strong user interaction through comments) were also included as data sources. All collected data from these Chinese social media platforms and websites were in Chinese. The data collection period spanned from November 2017 to December 2023, yielding a total of 17,880 comments. Specifically, the dataset comprises approximately 975 (5.45%) comments from Food Partner Network, 903 (5.05%) from Catering Industry Forum, 7,958 (44.51%) from Weibo, 4,021 (22.49%) from Zhihu, and 4,023 (22.50%) from Bilibili. This initial stage of data collection is crucial for capturing the raw, authentic consumer discourse that forms the basis for subsequent SMA steps.

### 2.2 Data preprocessing and cleaning

To enhance the quality of the data, irrelevant information was removed to ensure the accuracy and reliability of the data used in the study. We deleted posts from unverified individuals on Zhihu, Bilibili, and Weibo, and filtered out duplicate text and introductory content about future foods from the relevant websites. Additionally, meaningless and frequently repeated content from users was excluded. This preprocessing resulted in a valuable dataset of 10,992 comments to be used as the data source.

### 2.3 Topic modeling

#### 2.3.1 LDA framework

**Figure 2:**
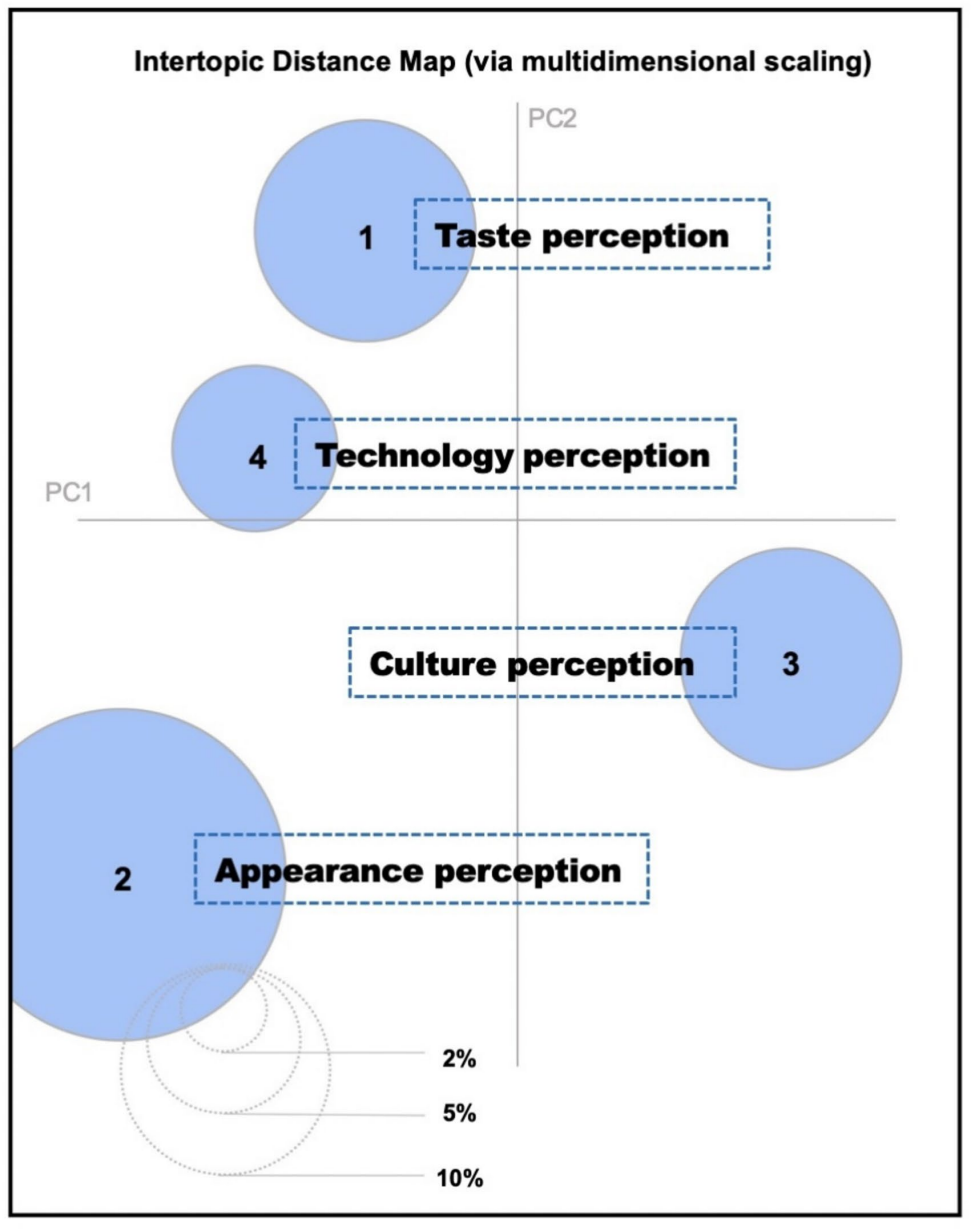
The intertopic distance map of the four LDA perceptions.

As part of the analysis stage within our SMA framework, Latent Dirichlet Allocation (LDA) is one of the classic algorithms in topic modeling. Proposed by (48) the LDA topic model is a probabilistic model for discrete data such as text corpora, and it is an unsupervised machine learning algorithm (27, 28). The LDA topic model is structured into three layers: texts, topics, and keywords, and is used to obtain the latent topic distribution within documents (44). The process of topic extraction is illustrated in the Figure 2 provided. In the figure, α represents the prior distribution of $\theta_{m}$ , where $\theta_{m}$ is the topic distribution for document m , $z_{m,n}$ is the topic for the n th word in document m , drawn from $\theta_{m}$ , and $w_{m,n}$ is the n th word in document m generated from topic $z_{m,n}$ . $\Phi_{z_{m,n}}$ is the word distribution, β is the prior distribution for the word distribution, and $N_{m}$ is the total number of words in document m , with a total of M documents. Each word $w_{m,n}$ in document m is generated by first drawing a topic $z_{m,n}$ from the topic distribution $\theta_{m}$ for that document, and then drawing a word from the corresponding word distribution $\Phi_{z_{m,n}}$ for that topic (29, 30).

#### 2.3.2 Process of topic modeling using LDA

After preprocessing the media text, the steps for topic modeling using the LDA method. First, individual word tokens are converted into vector representations for further computational processing. Next, the LDA model is developed using the Genism topic modeling package due to its scalability and convenience for processing text corpora related to future foods. Specifically, we employed the LdaModel class from the gensim. Models module. The number of topics (k) was determined through iterative testing and visualization using LDAvis, as described below, ultimately set to 4. The alpha and beta parameters, representing the prior distributions for topic-document and word-topic distributions respectively, were set to ‘auto’ to allow the model to learn asymmetric priors from the data. The number of training passes was set to 1,000, and the update every parameter was set to 1 for online learning. During the LDA visualization process, the interactive visualization system LDAvis is used to identify topic numbers and visualize the topics. Through LDAvis, the frequency and relevance of topics can be determined (31), and the number of topics is adjusted until there is sufficient separation between different clusters (31). Finally, the topic for each document is identified, with the topic probability distribution for each document outputted by the LDA model. Topics are determined based on their frequency. SnowNLP is applied to analyze the sentiment orientation toward future foods. Using the SnowNLP model, each text is assigned an emotion value ranging from 0 to 1, where 0–0.4 indicates a negative sentiment orientation, 0.4–0.6 indicates a neutral sentiment orientation, and 0.6–1 indicates a positive sentiment orientation.

#### 2.3.3 Word embedding analysis

Following the analytical process of word embedding, this analysis stage of our SMA framework employs the Word2Vec language model developed by Mikolov et al. (32) to represent the vocabulary in the text as high-dimensional vectors. To extract information related to the perception of future foods from textual content, we utilized natural language processing techniques known as “word embedding” to quantify the relationships between socio-psychological variables and conducted a cross-temporal comparison. The study used corpus data from 2017 to 2023. The word embedding model was trained on a large-scale text dataset, with the word vector dimension set to 200 dimensions. The SGN feature learning algorithm was employed, setting the context window for observing the textual environment of each word to 10 words before and after. Specifically, we used the Skip-gram model with hierarchical softmax as the training objective. The minimum word count was set to 5 to ignore words with very low frequency. The number of negative samples was set to 0 as we used hierarchical softmax. The number of worker threads was set to 4. The model was tested using the Wordsim-297 dataset, and the correlation between the word embedding model and human evaluation was found to be significant (*r* = 0.56, *p* < 0.001), indicating that the model training was effective.

#### 2.3.4 Sentiment analysis

Sentiment analysis is used to identify consumer attitudes and emotions toward future foods from textual data. Within our SMA framework’s analysis stage, there are two primary approaches to sentiment analysis. Given that unsupervised methods integrate emotion lexicons and rules, making them suitable for identifying sentiment orientation in fine-grained text (31, 46), this study adopts an unsupervised approach.

To monitor the sentiment of user comments online, this study matched sentiment lexicons with words in the text and calculated the sentiment orientation of the compared words. TF-IDF (Term Frequency-Inverse Document Frequency) and WordCloud were used to visualize the text within different comments. The simplified Chinese sentiment lexicon from National Taiwan University (NTUSD) was utilized in the R software to perform sentiment lexicon matching analysis on the preprocessed comment data. This allowed for determining the sentiment orientation of the user comments, calculating specific sentiment values and directions, and conducting statistical analysis on the sentiment orientation of the comments. Specifically, for each comment, we calculated a sentiment score based on the presence and weighting of words in the NTUSD lexicon. Positive words contributed positively to the score, while negative words contributed negatively. Neutral words were assigned a score of zero. The overall sentiment score for a comment was then normalized to a value between 0 and 1, where values closer to 1 indicate stronger positive sentiment, values closer to 0 indicate stronger negative sentiment, and values around 0.5 indicate neutral sentiment. The thresholds of 0–0.4 for negative, 0.4–0.6 for neutral, and 0.6–1 for positive sentiment orientation were set based on common practice in sentiment analysis using lexicon-based methods.

## 3 Results

### 3.1 Clustering of future food topics

LDAvis was used to explore the clustering results. In the analysis of future foods, four topics were identified, with the clusters being distinct and showing minimal overlap. Ultimately, four potential perceptual dimensions of future foods were identified. Figure 2 illustrates the distance between the themes of the four perceptual dimensions. To ensure the interpretability of the themes, 10 related texts were identified. By combining the relevant texts and representative posts with high probability scores under each dimension, the content was summarized into four main dimensions: taste perception, appearance perception, cultural perception, and technological perception of future foods. As shown in Figure 2.

To provide further clarity on the composition of these perceptual dimensions, we present representative sample words and concepts associated with each cluster, derived from the high-probability terms within each LDA topic.

To enhance the transparency and provide a clear rationale for the naming of these perceptual dimensions, we further examined the content of representative texts and the distribution of high-probability terms within each identified LDA topic. We present a description of each dimension, including illustrative sample text snippets and the key terms that informed our interpretation:

Taste perception: this dimension encompasses discussions related to the flavor, texture, and overall palatability of future foods. High-probability terms in this topic include: flavor, texture, delicious, taste, sweet, bitter, salty, savory, mouthfeel, palatability. Representative text snippets from this cluster often express opinions on the sensory experience, such as: “I’m curious about the taste of artificial meat, will it be like real meat?” or “The texture of this plant-based burger was strange, not like traditional meat at all.” The prevalence of terms directly describing sensory attributes and subjective eating experiences led to the labeling of this theme as “Taste Perception.”

Appearance perception: this dimension focuses on the visual aspects of future foods, including their color, shape, form, and presentation. High-probability terms in this topic include: color, shape, look, visual, appealing, unattractive, presentation. Examples of social media posts belonging to this cluster are: “The color of this lab-grown chicken looks a bit pale, not very appetizing,” or “The packaging of future foods needs to be more attractive to consumers.” The emphasis on visual characteristics and aesthetics in these discussions informed the “Appearance Perception” theme.

Cultural perception: this dimension reflects discussions about the socio-cultural implications of future foods, including their connection to tradition, cultural norms, identity, and social acceptance. High-probability terms in this topic include: tradition, culture, custom, heritage, acceptance, social norm, identity, local, global, food habits. Illustrative text snippets from this cluster include comments like: “Eating insects is against our traditional food culture,” or “Will future foods replace our traditional dishes? It feels like a challenge to our heritage.” The recurring themes of cultural values, traditions, and social acceptance guided the naming of this dimension as “Cultural Perception.”

Technological perception: this dimension pertains to discussions surrounding the technology used in the production of future foods, including aspects of science, innovation, safety, and process. High-probability terms in this topic include: technology, science, innovation, production, lab, gene, artificial, safety, process, future. Representative posts in this cluster often discuss the scientific advancements or potential risks, such as: “I’m amazed by the technology behind lab-grown meat, it’s truly innovative,” or “I’m worried about the long-term health effects of gene-edited food.” The focus on the scientific and technical aspects of future food production led to the designation of this theme as “Technological Perception.”

### 3.2 Relationship between future food neophobia and perceptual dimensions

In this study, a word list was constructed to extract five food evaluation dimensions through the following steps. Initially, a preliminary word list for the happiness perception dimension was established by consulting food scales, other survey scales, thesauri, and definitions provided by previous scholars. After filtering the word list, the general process for constructing word lists in the field was followed (33). The final candidate word list was confirmed after review by three experts.

The relationship between the perceptual dimensions of future foods and “food” evaluation was measured using cosine similarity to calculate vector distances. The study interprets the trends in each future food perceptual dimension, with the original calculation results based on the word embedding method illustrated in the figure provided. The relevance calculation is shown in Table 1.

**Table 1:** Illustration of semantic relevance calculation between neophobia and perception of future foods.

Cosine similarity		Perception of future food				
		Taste	Appearance	Culture	Technology	…
The concept of food neophobic	Fearful	0.3	0.4	0.5	0.5	…
	Degusted	0.5	0.6	0.5	0.3	…
	Exclusive	0.4	0.6	0.4	0.4	…
	Unnatural	0.4	0.5	0.6	0.4	…
	Risky	0.3	0.4	0.3	0.3	…
	…	…	…	…	…	…

**Figure 3:**
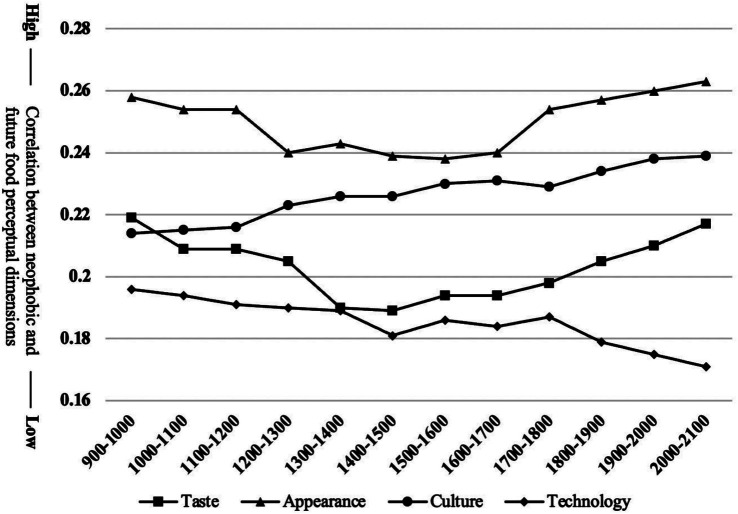
Trend of the relationship between neophobia and perception with increasing perceptual intensity.

This study conducted a quarterly analysis of the word frequency related to future food perception dimensions. Semantic relevance between neophobia behavior and future food perception dimensions was calculated based on these frequencies. Each perception dimension was then subjected to a neophobia state test at varying levels of perceptual intensity to explore the relationship between future food neophobia and the intensity of future food perception (Figure 3).

Overall, the perception of appearance in future foods has the most significant impact on neophobia behavior (*M* = 0.312, SD = 0.094, *p* < 0.001), followed by “cultural perception” (*M* = 0.218, SD = 0.079) and “taste perception” (*M* = 0.198, SD = 0.092). The perception of technology in future foods has the least impact (*M* = 0.189, SD = 0.082). Over the past 6 years, there have been significant changes in the four perceptual dimensions of future foods (*F* = 2.15, *p* = 0.029).

### 3.3 Public sentiment and perceptions of future foods

**Figure 4:**
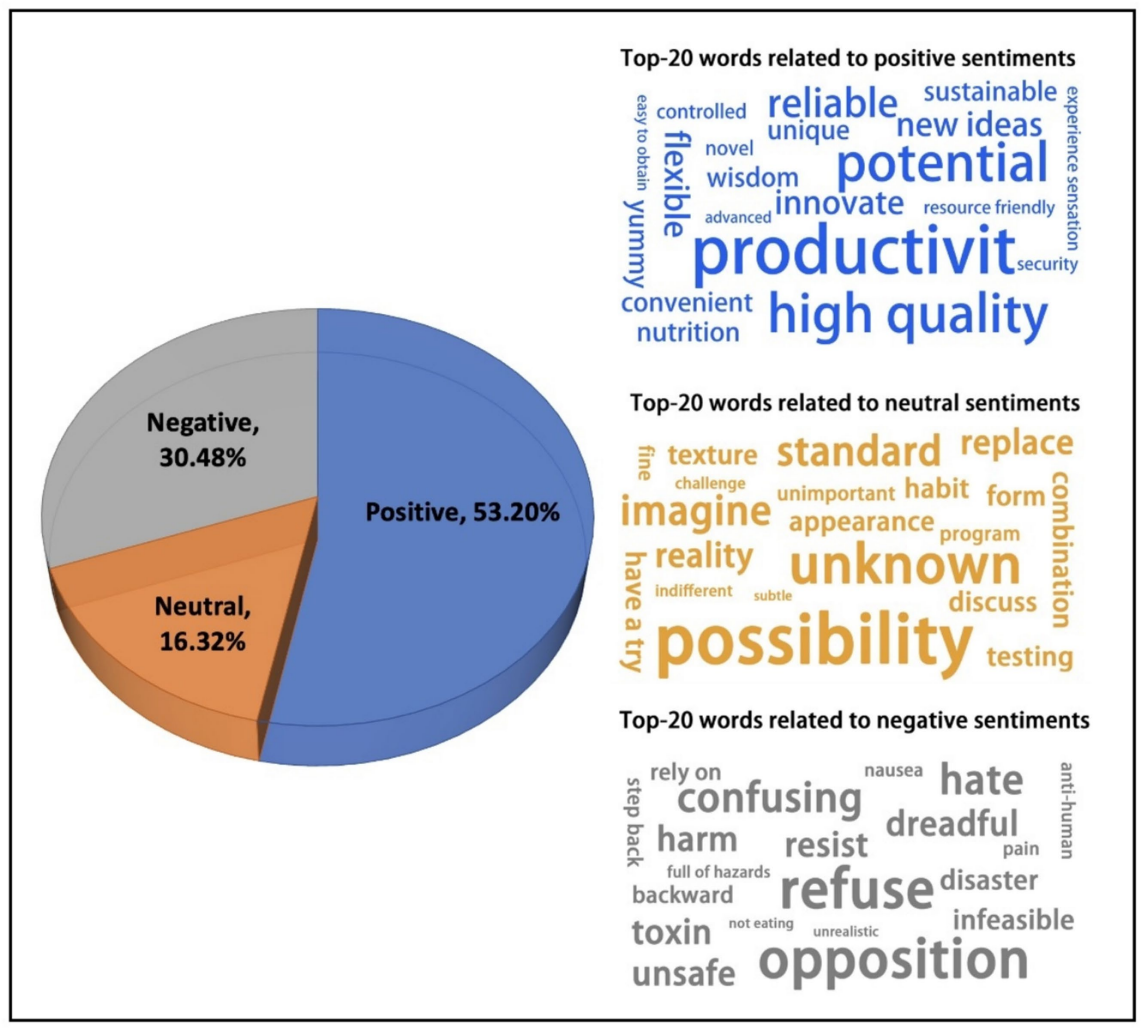
Sentiment distribution of text and the top 20 related words of each sentiment.

Public sentiment toward future foods was analyzed through the textual content related to future foods. The TF-IDF algorithm was used in this study to extract important keywords. As shown in Figure 4, the overall sentiment evaluation revealed that positive sentiment accounted for 53.20%, neutral sentiment for 16.32%, and negative sentiment for 30.48%.

### 3.4 Public perceptions of future foods across different dimensions

To clearly understand the emotional evaluations of consumers across different dimensions, and to identify which dimensions are more likely to elicit negative emotions, we conducted sentiment analysis for each of the four dimensions of future foods. The results are shown in Table 2.

**Table 2:** Emotional evaluation across different perceptual dimensions of future foods.

Perceptual dimension of future foods	Proportion of positive evaluations	Proportion of neutral evaluations	Proportion of negative evaluations
Taste perception	40.13%	18.43%	41.44%
Appearance perception	30.54%	20.98%	48.48%
Cultural perception	10.54%	30.76%	58.7%
Technological perception	40.65%	30.52%	28.83%

## 4 Discussion

### 4.1 Future food perception

The research on future food perception dimensions in this article explores which dimensions are important for future food perception, the results indicate that our study is consistent with Koirala et al.’s and Ibarra et al.’ research. Beyond confirming the relevance of taste, appearance, and cultural factors as highlighted in previous work (34, 35), our analysis of social media discourse further underscores the prominence of technological perception as a distinct dimension in shaping public attitudes toward future foods. This finding expands the traditional understanding of food perception by highlighting the increasing role of production technology in consumer evaluation, particularly in the context of novel food innovations.

Taste perception of future foods is characterized by its specificity and variability (36). The taste perception of future foods by consumers largely determines the competitiveness of the food, and the pleasure derived from the taste of future foods may offer consumers a higher level of satisfaction. Given the significant taste differences between future foods and traditional foods, taste perception emerges as a crucial dimension in the evaluation of future foods. This aligns with extensive research in consumer behavior demonstrating the fundamental role of sensory experience in food acceptance across various food categories.

The appearance of future foods can alter consumers’ basic perceptions of these foods. Food shape, including visual appearance, texture, volume characteristics, and packaging, plays a significant role in shaping consumers’ first impressions, which influences their judgments about product quality and, consequently, their acceptance of the food (47). Therefore, appearance perception is another critical dimension in the perception of future foods. Our findings on social media further emphasize the visual nature of initial impressions, particularly in a digitally-driven environment where visual presentation is paramount.

Food culture acts as a cultural dynamic that influences fundamental nutritional needs and food preferences across different ethnic groups (19). Consumers’ perceptions of future foods are deeply rooted in regional and cultural differences (37, 38), making cultural perception a vital dimension in the evaluation of future foods. The strong presence of cultural discourse in our social media data highlights how novel foods challenge established food norms and traditions, resonating with the growing body of literature on the socio-cultural determinants of food choice.

Technological perception of future foods involves the innovation and application of new technologies to enhance food safety, nutrition, and sustainable production (39). Consumers can experience the technological aspects of future foods during consumption, and therefore, technological perception serves as an essential dimension in the evaluation of future foods. Our identification of technological perception as a distinct dimension on social media suggests that consumers are actively engaging with the underlying technologies of future foods, reflecting a growing awareness and potential concern regarding their implications.

### 4.2 Future food perception and neophobia

The relationship between future food perception and neophobic behavior suggests that varying degrees of perception lead to non-linear changes in neophobic behavior (40). Our findings underscore the critical role of specific perceptual dimensions in triggering or mitigating neophobic responses, offering valuable insights for targeted interventions to enhance acceptance.

An increase in consumers’ taste perception level of future foods initially leads to a decrease in neophobia behavior, followed by an increase. This suggests that introducing taste novelty to consumers initially reduces neophobia, but as the intensity of taste stimuli increases, consumers may begin to worry that the new sensory technologies used in future foods could lead to perceptual distortions. This could manifest as abnormalities in sensory elements like taste and texture, raising concerns about the authenticity of flavors and resulting in what can be termed as the “taste novelty overload effect.” Our findings underscore the critical role of specific perceptual dimensions in triggering or mitigating neophobic responses, offering valuable insights for targeted interventions to enhance acceptance.

Regarding the appearance perception of future foods, as the perception level increases, consumers’ neophobia exhibits an inverted “U” shape relationship. There is an “appearance attractiveness reversal effect” (10), where initially, as the attractiveness of future food appearance increases, consumers’ perception of attractiveness also increases, reducing neophobia. However, as attractiveness continues to rise, consumers might feel discomfort due to potential design-induced attractiveness reversal. This finding is particularly relevant for product design and packaging, suggesting that while visual appeal is important, there’s a limit to how “unconventional” an appearance can be before triggering discomfort and neophobia.

In the context of cultural perception, an increase in cultural perception is associated with heightened neophobia. The culture represented by future foods can reflect consumer identity, social status (41), and social interactions (42). Food plays a significant role in culture, and when future foods exhibit a high level of cultural perception, consumers may feel that future foods challenge established customs, leading to increased neophobia. This highlights the deep-seated connection between food and cultural identity, emphasizing the need for future food initiatives to consider and potentially integrate with existing cultural food norms rather than appearing as a complete disruption. This observed increase in neophobia with higher cultural perception within our sample, which likely includes individuals with a strong connection to traditional food culture, aligns with the idea that future foods are perceived as a departure from established norms, triggering resistance among those who value culinary heritage.

As the technological perception of future foods increases, neophobia behavior decreases. This finding, particularly the downward trend of neophobia as technological perception intensifies, might be partially explained by the characteristics of our data source, which likely includes a significant proportion of individuals who are more informed or interested in the technological aspects of food. For these “food enthusiasts” or individuals with a high level of engagement with future food discussions, a deeper understanding of the technology behind future foods may actually alleviate concerns and reduce neophobia, as they perceive technology as a factor that enhances safety, efficiency, or innovation, rather than an unknown or risky element. This contrasts with potentially higher levels of technophobia or distrust in technology among the general public, where increased technological awareness might lead to greater apprehension. This may be due to consumers’ better understanding of the role of technology in ensuring food safety and the ability of technology to help producers better manage consumption risks (43), thereby enhancing the perceived transparency of future food technologies. This suggests that clear and transparent communication about the technology behind future foods can help build trust and reduce neophobia, aligning with research on the importance of transparency in fostering acceptance of novel technologies.

### 4.3 Future food sentiment analysis

Our research on the emotional evaluation of future food indicates that the overall perception of future food is highly positive, also include negative evaluation contents. Provided us with an overall understanding of future food evaluation. While the overall sentiment toward future foods on social media is positive, the substantial proportion of negative and neutral sentiments warrants closer examination. These negative sentiments are likely shaped by a combination of factors, including media coverage, the spread of misinformation, and deeply ingrained cultural attitudes toward food.

In the context of positive evaluations, keywords included “novel,” “unique,” “innovative,” “higher quality,” “reliable,” and “potential.” These keywords emerged from text segments filled with positive sentiment, such as “Future foods are truly unique, I’m willing to try them,” and “Future foods represent the future of food production and have great potential.” These positive expressions often reflect an optimistic outlook on the potential benefits of future foods, likely influenced by positive media portrayals highlighting innovation and sustainability.

In the realm of neutral sentiment regarding the perception of future foods, keywords included “possibility,” “try,” “test,” and “average.” These words were derived from statements like “I lack sufficient knowledge about future foods,” and “The legal regulations and quality control of future foods differ from traditional foods.” People with a neutral attitude generally maintain an objective and cautious approach toward future foods, showing no significant preference for or against them, often due to a lack of sufficient knowledge. This highlights the role of information gaps and the need for clear, accessible information to move consumers from a neutral stance toward acceptance.

Negative sentiment comments on future foods included keywords such as “unsafe,” “harmful,” “tasteless,” “ugly,” “smelly,” and “strange.” These words reflect people’s concerns about potential negative aspects of future foods, expressing negative emotions across multiple perceptual dimensions. Additionally, we found that the emergence of negative sentiment is often linked to sudden food safety issues. When a food-related problem arises, the frequency of negative keywords associated with future foods tends to increase rapidly. These negative sentiments are likely exacerbated by misinformation circulating online and deeply rooted cultural preferences for traditional, familiar foods. The observed spike in negative sentiment linked to food safety issues underscores the fragility of public trust and the significant impact of negative events, which can be amplified by social media.

### 4.4 Sentiment analysis in various perception dimensions

The results of different future food perception sentiment analysis indicate that appearance perception and cultural perception of future foods receive lower levels of positive evaluation. This outcome aligns with the observed relationship between these dimensions and neophobia behavior, confirming that the appearance and cultural perception of future foods have a more significant negative impact on consumption and acceptance. Food producers should pay particular attention to the appearance and cultural attributes of future foods when considering consumer acceptance. On the other hand, taste perception and technological perception of future foods show higher levels of positive impact, suggesting that future foods that exhibit better taste meet consumer expectations, and that advanced technology offers consumers low-risk and high-safety food options. These findings provide further support for the notion that appearance and cultural factors are significant psychological barriers to future food acceptance, as they elicit stronger negative emotional responses compared to taste and technology. This reinforces the importance of addressing the psychological roots of neophobia in marketing and communication strategies.

### 4.5 Practical implications

This study offers practical insights for the development, marketing, and regulation of future foods. Findings suggest that producers should strategically address consumer psychological barriers, particularly the strong influence of appearance and cultural perception on neophobia and negative sentiment. Enhancing the visual appeal and cultural alignment of future food products is crucial for fostering acceptance. Furthermore, clear and transparent communication about the underlying technology, emphasizing innovation, quality, and safety, can help build trust and mitigate concerns. These strategies are essential for facilitating consumer acceptance and promoting the successful integration of future foods into dietary practices.

### 4.6 Limitations and future research

While providing valuable insights, this study has limitations. The data are primarily derived from Chinese social media platforms, potentially limiting the generalizability of findings to other geographic and cultural contexts. Future research could expand data collection to include diverse platforms for cross-cultural comparisons. Additionally, relying solely on social media data may not capture the full spectrum of public opinion, suggesting the value of complementing this approach with traditional methods like surveys. Users who actively participate in discussions about future foods on these platforms, particularly those using specific hashtags like #FutureFoods#, may represent a segment of the population with a higher level of interest, prior knowledge, or specific opinions regarding novel food technologies and products compared to the general public. Future work should also explore alternative analytical techniques to uncover further nuances in perception and investigate the dynamic and causal relationships between perception, neophobia, and emotional sentiment over time.

## 5 Conclusion

This study, leveraging social media analytics, provides a novel and ecologically valid investigation into public perception and attitudes toward future foods, offering significant insights for both food science and psychology. Our findings delineate four key perceptual dimensions–appearance, taste, culture, and technology – that shape public understanding and acceptance of future foods, thereby extending the application of perceptual theory in the novel food context. Crucially, we reveal a nuanced and non-linear relationship between these perceptual dimensions and food neophobia, demonstrating that the intensity of sensory and cultural information significantly modulates neophobic responses, highlighting complex cognitive-emotional interactions underlying food choices. Sentiment analysis of social media discourse further indicates that while positive sentiment prevails (53.20%), negative sentiments (30.48%) are disproportionately concentrated within appearance and cultural perception dimensions. This emphasizes the pivotal role of psychological factors, particularly visual and cultural framing, in shaping initial aversion to novel foods, informing targeted psychological interventions to mitigate neophobia and promote acceptance. Practically, these results underscore the necessity for future food industries to strategically address consumer psychological barriers, focusing on enhancing the visual appeal and cultural alignment of future foods alongside technological advancements to foster broader public acceptance and pave the way for sustainable dietary transitions. From a theoretical perspective, this study contributes to the psychology of food choice by demonstrating the interplay of cognitive appraisals (perceptual dimensions), emotional responses (sentiment), and behavioral tendencies (neophobia), offering a framework for understanding consumer adoption of radical food innovations.

## Data Availability

The raw data supporting the conclusions of this article will be made available by the authors, without undue reservation.
